# Plant Responses to Nanoparticle Stress

**DOI:** 10.3390/ijms161125980

**Published:** 2015-11-06

**Authors:** Zahed Hossain, Ghazala Mustafa, Setsuko Komatsu

**Affiliations:** 1 Department of Botany, University of Kalyani, Kalyani 741235, West Bengal, India; 2 National Institute of Crop Science, National Agriculture and Food Research Organization, Tsukuba 305-8518, Japan; ghazalamustafa@affrc.go.jp

**Keywords:** nanoparticles, oxidative stress, proteomics

## Abstract

With the rapid advancement in nanotechnology, release of nanoscale materials into the environment is inevitable. Such contamination may negatively influence the functioning of the ecosystems. Many manufactured nanoparticles (NPs) contain heavy metals, which can cause soil and water contamination. Proteomic techniques have contributed substantially in understanding the molecular mechanisms of plant responses against various stresses by providing a link between gene expression and cell metabolism. As the coding regions of genome are responsible for plant adaptation to adverse conditions, protein signatures provide insights into the phytotoxicity of NPs at proteome level. This review summarizes the recent contributions of plant proteomic research to elaborate the complex molecular pathways of plant response to NPs stress.

## 1. Introduction

Nanotechnology is an emerging multidisciplinary field with a wide range of applications in cancer therapy, targeted drug delivery, electronics, cosmetic industry, and biosensors [1]. Nevertheless, unspecified release of metal-based nanoparticles (NPs) into the ecosystem has raised global concern about their potential phytotoxic effects. The NPs are extremely fine particles with lengths between 1 and 100 nm in at least two of their dimensions [2]. These are in fact intermediate in size between molecules and bulk materials. Division of bulk materials into smaller and smaller pieces gives them very unique physical and chemical properties [3]. Moreover, a high surface-to-volume ratio renders these nanoscale materials highly reactive or catalytic.

Bulk production of NPs often leads to their indiscriminate release in nature through industrial waste-waters [4,5]. A majority of the manufactured NPs contain heavy metals. Thus, soil and water contamination with metallic NPs has become an important environmental issue. Nanoparticles interact with the plants and results in the uptake and accumulation that affect their fate and transport in the ecosystem. Moreover, NPs could remain attached to the plant surface and impart physical and chemical damage to the plant organs. Usually, NPs enter the plant root system through the lateral root junctions and reach the xylem through the cortex and the pericycle [6]. Notably, NPs’ entry into the plant can be stopped by the cell wall. The specific properties of cell wall allowing the transport of NPs could be attributed to the pore size of cell wall [7]. The NPs that are in the size range within the cell wall pore size could effectively cross the cell wall and reach the plasma membrane [8]. The rate of entry depends on the size and surface properties of NPs. Indeed*,* the smaller NPs can enter into plant cells easily. In contrast, larger NPs, being unable to enter the cells, cannot affect the cell metabolic pathways [9]. Larger NPs can only penetrate through the hydathodes, flower stigmas, and stomata. Mechanism of interaction between NPs and plants could be chemical or physical. Chemical interactions involve the production of reactive oxygen species [1], disturbance of ion cell membrane transport activity [10], oxidative damage [11], and lipid peroxidation [12]. Following entry into the plant cells, NPs after mixing behave as metal ions and react with sulfhydryl, carboxyl groups and ultimately alter the protein activity. However, while conducting engineered nanomaterials (ENMs) mediated ecotoxicity study, much attention needs to be paid towards various artifacts which often lead to misinterpretations of results [13]. These potential factors include toxic impurities in ENM materials, their proper storage and dispersion in testing medium. Moreover, ENMs exert indirect toxicity which affects plant growth and development through nutrient depletion with passage of time, and estimation of ENM dispersal in organisms. In addition, ENMs face different changes (viz. settling, dissolution, agglomeration, *etc.*) during the exposure period, which is difficult to measure accurately. Due to increased surface area and properties, ENMs readily adsorb organic molecules and inorganic ions from the nutrient medium resulting indirect toxicity symptoms including chlorosis and wilting [14,15]. Moreover, during ENM exposure, organic acid in plant root exudates decreases the pH of the media, thus altering nutrient supply and ENM properties [16]. Inefficiency to explore the influence of these factors can direct to an inappropriate explanation of phytotoxicity and ultimately a fabricated impact of ENMs [13].

## 2. Plant Response to Nanoparticle Stress

NPs with different composition, size, and concentration, physical/chemical properties have been reported to influence growth and development of various plant species with both positive and negative effects [17]. Khodakovskaya *et al.* [18] reported that multi-walled carbon nanotubes markedly influenced tomato seed germination and seedling growth by up-regulating stress-related gene expression. In *Arabidopsis*, Al_2_O_3_-NPs were reported to be least toxic compared to zinc oxide, iron oxide, and silicon oxide nanoparticles [19]. Previous study highlighted the toxic effects of NPs on algae [20]. NPs like titanium oxide, zinc oxide, cerium oxide, and silver NPs were deposited on the surface of cell as well as in the organelles, which resulted in oxidative stress to the cell through the induction of oxidative stress signaling [21]. In *Cucurbita pepo*, the effect of silver, copper (Cu), zinc oxide, and silicon nanoparticles indicated that seed germination was unaffected by these NPs and their counterpart bulk materials; however, Cu nanoparticles reduced root length compared to the control and plants treated with the bulk Cu powder [22]. In rice, ZnO NPs, but not titanium oxide cause deleterious effects on the root length at early growth stages [23]. Riahi-Madvar *et al.* [24] indicated that the root growth of *Triticum aestivum* was affected by different concentrations of the alumina-nanoparticles; however, NPs did not affect the seed germination, shoot length, and dry biomass. In rice seedlings, nano-CuO treatment led to an increase in activity of antioxidant enzymes and elevated MDA concentration [25]. A similar experiment on the nano-CuO modulated photosynthetic performance and antioxidative defense system in *Hordeum vulgare* demonstrated restriction in root and shoot growth with decreased photosynthetic performance index [26]. Moreover, nano-CuO mediated DNA damage and plant growth restriction were reported in radish (*Raphanus sativus*) and ryegrass (*Lolium perenne* and *Lolium rigidum*) [27]. Changes in enzyme activities, ascorbate and free thiol levels resulting in higher membrane damage and photosynthetic stress have been documented in shoots of germinating rice seedlings on exposure to very high concentration of cerium oxide NPs [28]. Generation of ROS and reactive nitrogen species and H_2_O_2_ upon exposure to Ag and ZnO engineered NPs on the duckweed (*Spirodela punctuta*) suggest that toxicity of Ag and ZnO-NPs predominantly caused by both the particulates and ionic forms [29].

Among the various metal NPs, much attention has been paid to Ag-NP owing to their characteristic physiochemical and biological properties compared to the massive bulk material [30]. The Ag-NPs have wide applications as an essential component in different products like household, food, and industries because of their bactericidal and fungicidal properties [31]. Compared to the silver-based compounds, Ag-NPs, with increased surface area available for microbe interaction, are reported to be more toxic to bacteria, fungi, and viruses. Like other metal ions, Ag-NPs can also induce oxidative stress in bacteria, animals, algae as well as higher plants [32]. However, the impact of Ag-NPs on plants largely depends on various factors such as plant species, growth stage of plant, composition and concentration of the nanoparticles, and the experimental setup (temperature, treatment period, media composition, and method of exposure, *etc.*) [33]. Nano-Ag is one of the most extensively studied NPs whose toxicology has been examined in various crops [22,32,34]. Although Ag-NPs exposure is reported to be detrimental for plant growth, some studies have demonstrated the growth-enhancing properties of Ag-NPs in *Brassica juncea* [35], *Eruca sativa* [33], wetland plants [36], and *Phaseolus vulgaris* and *Zea mays* [37]. An investigation by Kumari *et al.* [34] revealed chromotoxic effects of Ag-NPs on the mitotic cell division in root-tip cells of *Allium cepa.* Moreover, Ag-NPs interact with the membrane proteins and activate signaling pathways, that leads to inhibition of cell proliferation [38,39].

Perusal of all these nanotoxicity studies over the past decade reveals that plant response to NPs stress has been evaluated extensively in various crops largely *at physiological and biochemical levels*. Rather less focus has been given to the study of plant-NPs interface at transcript level (Table 1). Microarray*-*based gene expression analysis of *Arabidopsis thaliana* roots on exposure to ZnO-NPs, TiO_2_-NPs, and fullerene soot indicates that the underlying mechanisms of phytotoxicity are highly specific to the nanoparticle [40]. Khodakovskaya *et al.* [41] designed an advanced method by amalgamating genetic, photothermal, and photoacoustic strategies for highly sensitive detection of NPs in different parts of tomato plants, most importantly the reproductive organs. Total gene expression analysis of tomato leaves and roots exposed to carbon nanotubes (CNTs) revealed up-regulation in the stress and water channel-related genes. A separate study demonstrated selective root growth in maize upon exposure to single-walled carbon nanotubes (SWCNTs) [42]. Transcriptional analysis suggests that nanoparticle-root cell interaction selectively modulates gene expression in seminal roots, thus affecting relative root growth and development. Similar to transcriptome analysis, only limited numbers of studies have emphasized the effects of nanoparticles stress on plants at proteome level.

## 3. Modulation of Proteome Composition under Nanoparticle Stress

Over the past decade, phytotoxicity of Ag-NPs has been evaluated extensively in various crops, largely at morphological, physiological, and biochemical levels. However, only limited studies have emphasized the effects of Ag-NPs stress on plants at proteome level (Table 2). Recently, Mirzajani *et al.* [43] performed a gel-based proteomic study to understand the effects of Ag-NPs toxicity on *Oryza sativa.* The root proteome study revealed that Ag-NPs-responsive proteins were primarily associated with oxidative stress response pathway, Ca^2+^ regulation and signaling, transcription, protein degradation, cell wall synthesis, cell division, and apoptosis. Increased abundance of defense-related proteins including superoxide dismutase, l-ascorbate peroxidase, glutathione-S-transferase implies accelerated production of ROS under Ag-NPs treatment. It has been hypothesized that Ag-NPs or released ions impede cell metabolism by binding to second messenger calcium ion receptors, calcium ion channels, and Ca^2+^/Na^+^–ATPases.

**Figure 1:**
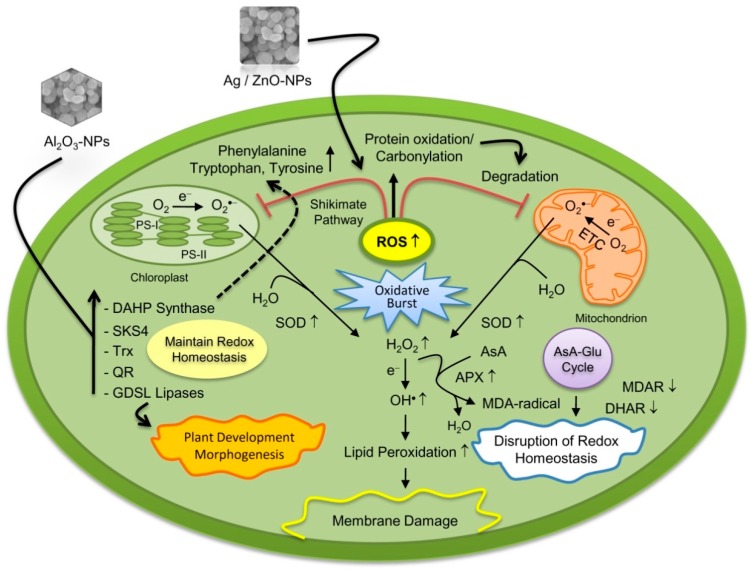
Cellular toxicity induced by nanoparticles (NPs). Exposure to NPs potentially leads to toxic side effects such as enhanced ROS generation, disruption of redox homeostasis, lipid peroxidation, impaired mitochondrial function, and membrane damage. Upward arrows indicate increased and downward arrows indicate decreased protein abundance in response to NPs stress, respectively. Dotted arrow represents shikimate pathway, a common biosynthetic route for the synthesis of aromatic amino acids. Abbreviations: APX, ascorbate peroxidase; AsA, reduced ascorbate; DAHP, 3-deoxy-*D*-arabino-heptulosonate-7-phosphate; DHAR, dehydroascorbate reductase; ETC, electron transport chain; H_2_O_2_, hydrogen peroxide; MDA, malondialdehyde; MDAR, monodehydroascorbate reductase; PS, photosystem; QR, quinone reductase; ROS, reactive oxygen species; SKS4, SKU5 similar 4 protein; SOD, superoxide dismutase; Trx, thioredoxin.

Proteomic study on *Eruca sativa* roots exposed to Ag-NPs and AgNO_3_ revealed that both forms of Ag caused changes in the proteins related to redox regulation, disrupting cellular homeostasis (Figure 1) [33]. However, the Ag-NPs alone were responsible to alter the ER and vacuolar proteins, thus indicating these organelles as target sites of Ag-NPs. These findings suggest that phytotoxicity of Ag-NPs is primarily due to their characteristic physiochemical properties, and not by releasing the Ag^+^ [33]. We also studied the toxicity mechanisms of Ag-NPs on early-stage-soybean growth under flooding stress [44]. In total, three different particle sizes (2, 15, and 50–80 nm) and concentrations (0.2, 2, and 20 ppm) were screened. The Ag-NPs of 15 nm facilitated the soybean growth under flooding, compared to the larger and smaller nanoparticles. The changed proteins under Ag-NPs exposure were mainly related to stress, signaling, and cell metabolism.

**Table 1:** Summary of gene expression analyses in response to nanoparticle stress.

Plant (Cultivar)	Tissue/Organ	Growth Stage	Nanoparticles (Particle Size, Dose)	Treatment Period	Technique	Major Findings	Ref.
*Arabidopsis thaliana* accession Columbia-0	Root	3-week-old	Ag (10–80 nm), TiO_2_ (10–40 nm), MWNT	7 days	Microarray analysis	Exposure to NPs repressed expression of phosphate-starvation and root-development genes.	[45]
*Arabidopsis thaliana* (ecotype, Columbia) Wild, *tir1*, *abi5* mutants	Seedling	4-day-old	Ag-NPs: triangular (47 nm), spherical (8 nm), decahedral (45 nm)	3 days	qRT-PCR	Ag-NPs induced ROS accumulation; interfered with ethylene biosynthesis; promoted root growth; triggered gene expression involved in cellular events-cell proliferation, metabolism, and hormone signaling pathways.	[46]
*Arabidopsis thaliana* (Col-0/Redei-L211497)	Seedling	Germinating	Ag-NPs (20 nm; 5 ppm)	10 days	Microarray analysis	Up-regulated genes primarily associated with metals and oxidative stress, while down-regulated genes linked to biotic and hormonal stimuli.	[47]
*Chlamydomonas* *reinhardtii* (wild-type strain C137)	–	–	Ag (20 nm; 1 ppm), TiO_2_(5 nm; 1 ppm), ZnO (20 nm; 1 ppm), QDs (6–10 nm, 0.12 ppm)	2 h	RNA-seq analysis	Genes associated with photosynthesis were markedly decreased on exposure to TiO_2_. Ag-NPs exposure led to the elevation of transcripts encoding components of cell wall and flagella.	[48]
*Arabidopsis thaliana* (wild type, cv. Columbia)	Root	3-week-old	ZnO (<100 nm; 100 ppm), TiO_2_ (<150 nM; 100 ppm), FS (100 ppm)	7 days	Microarray analysis	Both abiotic (oxidative, salt, water deprivation) and biotic (wounding and defense to pathogens) stress responsive genes were up-regulated under ZnO and FS; while cell organization and biogenesis associated genes were down-regulated upon ZnO-NPs.	[40]

Abbreviation: FS, fullerene soot; MWNT, multi-wall nanotubes; QDs, CdTe/CdS quantum dots.

**Table 2:** Summary of proteomic analyses in response to nanoparticle stress.

Plant (Cultivar)	Organ	Growth Stage	Nanoparticles (Particle Size, Dose)	Treatment Period	Technique	Major Findings	Ref.
Soybean (*Glycine max* L. cv. Enrei)	Root including hypocotyl	2-day-old	Ag, ZnO, Al_2_O_3_-NPs with flooding stress (0.5–500 ppm)	1–3 days	Gel-free (nanoLC MS/MS)	Al_2_O_3_-NPs (50 ppm) promote seedling growth under flooding stress by regulating energy metabolism and cell death.	[49]
Soybean (*Glycine max* L. cv. Enrei)	Root, cotyledon	2-day-old	Ag-NPs with flooding stress (2, 15, 50–80 nm; 0.2, 2, 20 ppm)	1–4 days	Gel-free (nanoLC MS/MS)	Ag-NPs (15 nm: 2 ppm) treatments facilitate seedling growth under flooding stress. Decreased abundance of glyoxalase II 3 and fermentation-related proteins: pyruvate decarboxylase 2 and alcohol dehydrogenase 1, indicating metabolic shift from fermentative pathways towards normal cellular processes.	[44]
Rice (*Oryza sativa* L. cv. IR651)	Root	10-day-old	Ag-NPs (30, 60 ppm)	20 days	Gel-based (2-DE, nanoLC/FT-ICR MS)	Increased abundance of proteins related to oxidative stress response pathway, Ca^2+^ regulation signaling, transcription, protein degradation, cell wall synthesis, cell division and apoptosis.	[43]
*Eruca sativa* (common name: rocket)	Root	Germinating seeds	Ag-NPs or AgNO_3_ (0.1, 1, 10, 20, 100 ppm)	5 days	Gel-based (2-DE, nanoLC-nESI-MS/MS)	Alteration of some proteins related to the ER and vacuole indicating these two organelles as targets of the Ag-NPs action. Effects of Ag-NPs are not simply due to the release of Ag^+^.	[33]

In roots and cotyledons the abundance of glyoxalase II 3, an important enzyme of glyoxalase detoxification pathway, was increased in a time-course manner under flooding stress; however, it declined in response to Ag-NPs. Furthermore, Ag-NPs treatment caused a metabolic shift from fermentative pathways towards normal cellular processes. The results suggested that the Ag-NPs (15 nm at 2 ppm) treated soybeans experienced less deprivation of oxygen, which acts as an important factor for enhanced growth of soybeans under Ag-NPs treatment with flooding stress. In contrast, high concentration of Ag-NPs (20 ppm, 15 nm particle size) was lethal to soybean seedlings [44].Very recently, we compared the effects of Ag-, ZnO- and Al_2_O_3_-NPs on two-day-old soybean under flooding stress [49]. Interestingly, enhanced soybean growth was observed in 50 ppm Al_2_O_3_-NPs treatment. The Al_2_O_3_-NPs-responsive proteins were predominantly related to protein synthesis/degradation, glycolysis, and lipid metabolism. Moreover, 5-fold enhanced abundance of NmrA-like negative transcriptional regulator family protein was recorded under Al_2_O_3_-NPs treatment. In summary, proteomic findings suggest that regulation of energy metabolism and reduced root cell death might promote soybean growth under flooding stress.

## 4. Conclusions and Future Prospects

By summarizing proteomic contributions, efforts have been made in the present review to delineate the molecular basis of acquisition of nanoparticles stress response mechanism. However, only limited numbers of proteomic studies have so far been conducted in the plant system. Most of the studies carried out so far primarily deal with the overall plant response towards a specific NPs stress showing differential abundance of proteins involved in oxidation-reduction, ROS detoxification, stress signaling, and hormonal pathways. Proteomic studies on Ag-NPs induced phytotoxicity revealed that the size of the nanoparticle is the key factor in determining the type and magnitude of the cellular response. Future initiatives need to be taken to find out whether the metallic nanoparticles exert their toxicity solely due to their unique properties or to the released metal ions. Moreover, research aimed at identifying and characterizing subcellular organelle proteins are expected for exploring the precise alterations in the protein signature of cell to withstand the NPs stress. In addition to proteomics, other “omics” based high-throughput techniques such as transcriptomics and metabolomics have immense potential to evaluate the effects and toxicity of nanoscale materials [45,50]. Metabolomics allows fast screening for biomarkers of oxidative stress following the application of NPs. Moreover, combination of NMR- and LC/MS-based metabolomics approach is being exploited to investigate the specific pathways of interest including those related to oxidative stress, an inevitable consequence of nanoparticle exposure [50]. MALDI MS imaging technique, a powerful tool for nanotoxicology study, provides a snapshot of how NPs are distributed in tissues, which is important for characterizing and understanding nanomaterial-based toxicity [51]. Furthermore, the plant’s response to combined NPs would be another topic for future “omics” based research that could highlight the possible interaction between stress signaling pathways. All these valuable information would further provide us an extensive and elaborated picture about the response mechanism of NPs stress in plants.
